# “Longing is good”: proof-of-concept for a novel psychological intervention to tackle self-blaming emotions

**DOI:** 10.3389/fpsyg.2024.1438896

**Published:** 2025-01-07

**Authors:** Nahed Lajmi, Suqian Duan, Jorge Moll, Roland Zahn

**Affiliations:** ^1^Department of Psychological Medicine, Centre for Affective Disorders, Institute of Psychiatry, Psychology and Neuroscience, King’s College London, London, United Kingdom; ^2^Cognitive and Behavioral Neuroscience Unit, D’Or Institute for Research and Education, Rio de Janeiro, Brazil; ^3^National Service for Affective Disorders, South London and Maudsley NHS Foundation Trust, London, United Kingdom

**Keywords:** depression, self-blame, guilt, sadness, intervention, psychotherapy, longing, digital health

## Abstract

**Background:**

Many people with depression, for which self-blame plays a key role, are not amenable to current standard psychological treatments. This calls for novel self-guided interventions, which require less attention and motivation. The present study sought to establish proof-of-concept for a novel self-guided intervention in a non-clinical sample, which prompts people to transform self-blaming feelings into “longing,” as a related unpleasant, but presumably more adaptive and approach-related emotion, which plays a key role in many musical and literary genres but has been largely overlooked in clinical research.

**Methods:**

Thirty nine participants with no previous self-reported history of mental disorders, but who experienced frequent feelings of self-blame were enrolled remotely (*n* = 3 lost to follow-up). Self-blaming thoughts, along with depressive symptoms and other outcomes, were assessed pre- and post-intervention to determine the potential of the intervention, which consisted of creating a 10-min video starting with self-blame evoking materials, transitioning to sadness and finally longing. Participants were then asked to watch their video daily for 7 days.

**Results:**

The number of participants lost to follow-up was low (*n* = 3) and engagement with the intervention was very high. As predicted, the intervention significantly reduced depressive symptoms on our primary outcome measure (Beck’s Depression Inventory, BDI-II, Cohen’s *d* = 0.40) after 1 week. This was further confirmed by a reduction on the Maudsley-modified Patient Health Questionnaire-9. Unexpectedly, no changes were observed on the non-clinical measures.

**Limitations:**

A randomized controlled trial design is needed to determine whether these symptom reductions were causally related to the intervention. Additionally, our findings may not generalize to men, due to our inability to recruit a sex-balanced sample.

**Conclusion:**

As predicted, participants benefited from being prompted to transform self-blaming feelings into those related to longing which shows the feasibility and potential to be further developed in people with clinical depression. Our study highlights the adaptive potential of unpleasant approach-related emotions such as longing, which are rarely considered in standard psychotherapies.

## 1 Introduction

Freud (1953) proposed that depression was distinguishable from healthy grieving by self-blaming and self-punishing feelings. Similarly, the revised learned helplessness model suggested that vulnerability to major depression is due to a propensity to blame oneself for failure and negative events in an excessive and over-generalized way (Abramson et al., 1978). This has been confirmed by several empirical studies, which found exaggerated self-blaming biases (Duan et al., 2023) even in patients who had remitted from major depression (Zahn et al., 2015) relative to negative emotions towards others. Self-blaming feelings were further shown to be experienced by over 80% of people with fully remitting forms of major depressive disorder (MDD) (Zahn et al., 2015). Importantly, 85% of participants stated that negative emotions towards themselves were the most burdensome, whilst only a minority, approximately 10%, were bothered by negative feelings towards others (Zahn et al., 2015).

Whereas it is clear that a strong link between self-blame and depression exists, less is known about the underlying causes and mechanisms of these overgeneralized feelings of blaming oneself. Psychoanalytic views proposed that self-blaming feelings stem from an unconscious wish to hurt others, which results in feelings of shame and self-blame for having such perverse and antisocial impulses (Freud, 1923, 1924, 1930; Klein, 1935, 1946). A contrasting perspective was put forward by Weiss (1993) in his Control-Mastery Theory (CMT). According to the CMT, guilt is a result of altruism and concern about others, thus, it is an interpersonal and adaptive emotion. In line with attachment theory (Bowlby, 1978), CMT is based on the idea that, during their early interactions, children develop an inclination to care for others and to take responsibility for their well-being. From infancy, children experience distress when exposed to others’ distress (Sagi and Hoffman, 1976) and by 16 months, they start to engage in behaviors aimed at relieving others’ distress. When unable to do this, they experience the antecedents of empathy-based guilt (Zahn-Waxler et al., 1992).

Although guilt can be deemed a healthy and adaptive feeling, as it contributes to the maintenance of relationships, it can become maladaptive when it is exaggerated, results in distress and disrupts a person’s functioning (Tangney et al., 1992; Tangney et al., 1995; Zahn-Waxler and Kochanska, 1988). As an example, exaggerated self-blame may stop a person from pursuing their 67 interests or goals, as they might believe that, by following their objectives, they could cause harm to 68 someone else. Research has, in fact, uncovered a link between excessive intrapersonal guilt feelings and psychological problems, along with self-defeating behaviors (Berghold and Lock, 2002; Bruno et al., 2009; Giammarco and Vernon, 2015).

Importantly, self-blaming emotions have often been linked to maladaptive action tendencies, such as ‘feeling like hiding’ (Haidt, 2003). Action tendencies are defined as a motivational and cognitive state in which there is an increased likelihood of engaging in certain behaviors. Although it was hypothesized that adaptive moral emotions were meant to promote constructive action tendencies, and maladaptive moral emotions resulted in dysfunctional action tendencies (Tangney et al., 2007), the actions that follow the experience of an emotion are likely to be context-dependent. In addition, individual differences in the likelihood of a particular emotion eliciting adaptive or maladaptive action tendencies are also at play. To illustrate, when experiencing guilt, one can respond by apologizing and making amends (adaptive action tendency) or by socially withdrawing (maladaptive action tendency). Duan et al. (2022) found that remitted MDD patients, compared to individuals with no history of depression, were more likely to exhibit maladaptive self-blame related action tendencies, such as hiding and creating a distance from themselves rather than taking reparative actions. This is in line with the finding that patients with MDD use more avoidance coping styles (Haskell et al., 2020).

In addition, according to the appraisal theory of emotions, which proposes that our reaction to emotional stimuli is context- and motive-dependent (Moors and Fischer, 2019; Montalti and Mirabella, 2023), depressed individuals present appraisal biases which facilitate the elicitation of negative emotions, such as self-blame, more frequently and intensely (Mehu and Scherer, 2015). Thus, depressed patients are not only likely to respond to negative emotions in a maladaptive way, they also tend to interpret events and situations in negative ways, thus, creating a vicious cycle in which they interpret and respond to emotional stimuli in unhelpful manners.

Studies have suggested that adaptive guilt differs from overgeneralized and excessive guilt, in that the former is usually relieved by either making reparations to the harm caused or by acknowledging one’s share of responsibility, whereas irrational and out of context guilt does not decrease following such actions (Regan, 1971). ‘Irrational’ and generalized guilt indeed seems more challenging to alleviate, given that it does not appear to be context-dependent or to result from actual responsibility.

CMT proposes four types of interpersonal guilt: survivor guilt, separation guilt, omnipotent responsibility guilt and self-hate (O’Connor et al., 1997). Survivor guilt refers to the guilt that people experience when they believe that, by following their goals and being successful or happy, they will inevitably harm others (Niederland, 1981). Separation guilt, on the other hand, was proposed to be due to a fear of hurting others by becoming independent or by holding different values or beliefs (O’Connor et al., 1997). Omnipotent responsibility guilt was thought to stem from an excessive sense of responsibility for the well-being of others. Finally, self-hate was thought to result from feelings of being inherently inadequate and not deserving of love; these are beliefs that the individual might have internalized following abusive or neglecting environments (O’Connor et al., 1997).

Given the extensive evidence on the role of self-blame in MDD, these self-blaming emotions and their elicited maladaptive actions tendencies could be potential treatment targets for psychological interventions. Dysfunctional thinking patterns, such as those exhibited by individuals who suffer from over-generalized blame towards themselves, are usually challenged by most common therapies, such as Cognitive Behavioral Therapy (CBT) and Mindfulness-based Cognitive Therapy. However, recovery from CBT for depression ranges between 32 and 43%, suggesting that over half of patients remain unwell. Perhaps more importantly, access to CBT remains challenging due to limited health care resources (Bower and Gilbody, 2005), and of those who are offered therapy, over 30% do not adhere (Barnes et al., 2013). Psychological therapies, such as CBT, require a high level of commitment and time from participants; they are, indeed, expected to attend several therapy sessions, engage with the therapist, complete homework in between sessions and increase their activity levels. However, when in a depressive episode, individuals tend to lack motivation, feel de-energized and lose hope and interest easily (Seime and Vickers, 2006). Thus, the lack of resources, as well as the large number of patients withdrawing from therapy, call for the development of less demanding and more flexible self-guided interventions.

Additionally, it is worth recognizing that MDD affects individuals’ ability to think, focus, make decisions, reason and form memories (Beck and Bredemeier, 2016; Everaert et al., 2012). Studies have documented the presence of memory biases in depression: patients not only exhibit a propensity to form and recall memories that are congruent to their mood state (Lloyd and Lishman, 1975), but they also seem to suffer from short-term memory impairments (Breslow et al., 1980; Steif et al., 1986). These memory problems are, however, partly due to other impairments, such as attentional difficulties (Marazziti et al., 2010). Therefore, patients would benefit from the development of novel interventions, which demand less overt attention and, ideally, take advantage of implicit memory. Such interventions could employ passive learning methods, such as priming or repetition, which, without requiring participants to intentionally focus on remembering, result in the formation of new memories through repeated exposure to stimuli (Ghuman et al., 2008).

Other passive learning methods that could be employed to help depressed individuals move away from a state of excessive and generalized blame include the use of mood induction. It is now recognized that the induction of different emotional states impacts patients’ answers on questionnaires, as well as their recalled memories (Lau et al., 2004). To illustrate, inducing a depressed mood in participants resulted in the recall of more negative words, whereas inducing an elated mood led to the recall of more positive words (Teasdale and Russell, 1983). Mood states can be elicited by exposing individuals to either music (Clark and Teasdale, 1985), retrieved memories (Brewer and Doughtie, 1980), self-referent mood statements, such as “I have too many bad things in my life,” (Velten, 1968) stories or film-clips (Marston et al., 1984).

Of particular relevance to the development of novel psychological interventions is a study conducted by Jaeckle et al. (2023), which demonstrated that, contrary to popular beliefs, actively thinking about, rather than suppressing, negative feelings, such as self-blame and guilt, does not give rise to increased depressive symptoms. Indeed, applying one simple self-guided strategy and repeatedly thinking about events associated by participants with self-blaming emotions, resulted in a decrease in depressive scores and an increase in self-worth. This is consistent with the finding that thought suppression is a counter-productive strategy, which often results in an enhanced frequency of intrusive thoughts (Wegner et al., 1987) and further highlights the damaging effect of avoidance / withdrawal action tendencies.

All of the above evidence provides alternative processes that could be employed in psychological therapies for the reduction of self-blame in MDD. Of significance is the fact that such interventions would rely on a lower level of attention and effort from patients. Importantly, previous unpublished pilot work from our lab attempted to test a novel psychological intervention, which makes use of repetition, emotion induction methods, as well as sequential transitions of emotions inspired by musical cadences (Chanturishvili and Zahn, 2018). Through daily exposure to a 10-min video clip for 7 days, the intervention aimed to sequentially transform participants’ feelings of self-blame into feelings of sadness, which entails no attribution of blame (Weiner, 1985). This resulted in a meaningful decrease in depressive symptoms.

Based on this previous project, the present study aimed to prompt people to implicitly transform feelings of sadness into longing. Longing, in this case, referred to a yearning desire for something in the past or something considered unattainable. Synonyms of longing were nostalgia, yearning desire, as well as appreciation and admiration for something that had been lost. Longing is a major theme of various musical genres, such as bossa nova (“saudade”), which prompted the development of the current intervention.

The reason why feelings of longing were induced, instead of happiness or joy, lies in the nature of MDD. While in a depressive state, individuals fail to find pleasure or happiness from their surrounding environment (American Psychiatric Association, 2013); they even struggle with recalling memories in which they were happy (Lloyd and Lishman, 1975). Prompting them to shift from self-blame to happiness is unlikely to be successful, in fact, in most cases, it would result in a deterioration of mood (Wegner et al., 1993). However, we hypothesized that shifting from self-blame to sadness and later to longing might be, not only more intuitive, as these feelings are all shades of negative emotions, but also beneficial. Sadness has, indeed, numerous adaptive functions (Horwitz and Wakefield, 2007) and its adaptive aspects have been repeatedly emphasized: it signals dissatisfaction, optimises information processing and motivates people to deal with difficulties (Bodenhausen et al., 1994). Longing, on the other hand, represents a sense of appreciation and adoration for memories or objects, which, in turn, reinstates desire and purpose. As an example, Sedikides and Wildschut (2018) proposed that nostalgia helps people find meaning in their lives, and it does so primarily by increasing social connectedness (a sense of belongingness and acceptance), and secondarily by augmenting self-continuity (a sense of connection between one’s past and one’s present).

The present study tested the proof-of-concept and safety of a novel online psychological intervention in a sample of participants with no diagnosed mental health problems, who reported that self-blaming emotions were a problem for them. Through the employment of induction methods, the intervention aimed to shift individuals from a state of self-blame to sadness, and later to longing. This was achieved by asking participants to create a 10-min film clip containing media materials, which they personally associated with self-blame, sadness and longing. As this represented a new intervention, it was deemed necessary to first investigate it in a non-clinical sample.

The study’s main hypotheses were:

1. After 1 week of the intervention, self-blaming emotions, as assessed through the Interpersonal Guilt Questionnaire (IGQ; O’Connor et al., 1997), will decrease (primary non-clinical outcome);
2. Similarly, depressive scores on the Beck Depression Inventory (BDI-II) will decrease post-intervention (primary clinical outcome).

This investigation further explored changes on secondary clinical, such as the Maudsley Modified PHQ-9, and non-clinical outcomes, including the Rosenberg Self-esteem scale, in order to identify the potential of these instruments as alternative outcome measures for future trials.

## 2 Methods

This present proof-of-concept trial was granted ethical approval by the King’s College London’s Research Ethics Committee, under the following reference: HR/DP-20/21–21,550. The study was fully conducted online and took place during the 2021 national lockdown and the subsequent local restrictions.

### 2.1 Participants

Thirty-six participants without any self-reported mental health diagnoses, recruited through online advertisements circulated on the university’s mailing list and on social media, voluntarily took part in this study. All subjects were aware of their withdrawal rights and gave informed consent to the storage and use of their anonymized data before participating in the trial.

In order to be enrolled in the study, participants were invited to fill in a pre-screening questionnaire on Microsoft Forms to ensure their eligibility. A total of 1,839 people completed the pre-screening survey. Inclusion criteria were as follows: participants with no previous history of mental or significant medical disorders, above the age of 18, fluent in English, with a predisposition to self-blame, demonstrated by a positive response to the question “*Do you often worry to have done something wrong?*.” This question was used to evaluate the presence of self-blame, as illustrated by its inclusion in a phenomenological psychopathology-based interview, and in the Maudsley Modified PHQ-9 (Zahn et al., 2015; Harrison et al., 2021). Participants were also asked to rate the frequency of these worries on a 7-point Likert scale, ranging from 0, *never*, to 7, *every day, most of the day*, to determine the severity of their self-blaming feelings.

Participants who met these eligibility criteria were invited to fill in the Patient Health Questionnaire 9 (PHQ-9) (Kroenke et al., 1999) and the Mood Disorders Questionnaire (MDQ) (Hirschfeld et al., 2000) to ensure that any potential manic or depressive symptoms were below the clinical cut-off scores. Accordingly, subjects who scored 10 or above on the PHQ-9, indicating clinically relevant depressive symptoms, were excluded. Participants were also not eligible to take part in the study if they expressed any suicidal ideation, as evidenced by a response different from “*never*” on item 9 of the PHQ-9, that is “*thoughts that you would be better off dead or of hurting yourself in some way*.” With regards to the MDQ, 7 or more of the listed symptoms, most of which occurring within the same time and causing moderate to severe problems to the individual’s functioning, would lead to exclusion.

These stringent inclusion criteria, that is having a tendency to self-blame, but not suffering from depressive symptoms, resulted in a markedly limited sample of suitable participants, specifically 187 subjects. Of these 187 eligible participants, thirty-nine were recruited, 3 of which dropped out throughout the study: one of these reported that participation was too time-consuming, whereas the other two did not give a specific reason. The remaining participants were 81% females (*Mean age* = 28.11 ± 10.12, *Range* = 18 to 62) and 97% had achieved at least an A-Level Qualification (see Table 1 for more detailed demographic characteristics). Their pre-screening PHQ-9 scores ranged from 0 to 9, with an average of 4.81 ± 2.46, whilst their MDQ scores ranged from 0 to 9, with an average of 2.28 ± 2.40.

**Table 1:** Summary of participants’ demographic characteristics.

Demographic characteristics	Number of participants
Sex at birth	
Female	19/36
Male	7/36
Ethnicity	
White	23/36
Mixed/Multiple ethnic groups	1/36
Asian/Asian British	9/36
Black (African, Caribbean, Black British)	1/36
Other ethnic group	1/36
Qualifications	
GCSEs or equivalent	1/36
A-levels or equivalent	15/36
Bachelor	7/36
Master’s	12/36
PhD	1/36

### 2.2 Materials

#### 2.2.1 Symptom measures and screening instruments

*The Interpersonal Guilt Questionnaire* (IGQ-67) (O’Connor et al., 1997) is a 67-item self-reported scale which assesses four different types of guilt conceptualized by the Control-Mastery Theory (Weiss, 1993): survivor guilt (22 items), omnipotent guilt (14 items), self-hate (14 items) and separations guilt (15 items). Items are rated on a 5-point Likert scale, ranging from 0, *very untrue of me*, to 5, *very true of me* and they include statements such as “*I deserve to be rejected by people*.” This scale’s validity and reliability has been established both in clinical and non-clinical samples (O’Connor et al., 1997).

*The Beck Depression Inventory, 2*nd *edition* (BDI-II) (Beck et al., 1996) is a 21-item self-rated scale designed to assess the bi-weekly presence and severity of depressive symptoms according to the diagnostic criteria listed on the Diagnostic Manual of Mental Disorders 4th edition (DSM-IV) (APA, 1994). Each of the items corresponds to a symptom of depression and is rated on a four-point scale ranging from 0 to 3. Total scores below 14 are deemed to be within the healthy range. The BDI-II has been reported to be highly reliable and it has been validated in numerous countries and across clinical and non-clinical samples aged 13 to 80 years (Lee et al., 2017; Rodríguez-Gómez et al., 2006; Sica and Ghisi, 2007).

*The Maudsley Modified Patient Health Questionnaire (MM-PHQ-9)* is a reliable and validated self-report measure of depressive symptoms. It was developed by Harrison et al. (2021) to address the standard PHQ-9’s weakness in accurately measuring change in depressive symptoms over time. The MM-PHQ-9 differs from the original PHQ-9 in that: (1) items regarding hopelessness and depressed mood were separated, as the latter takes longer to improve during treatment; (2) somatic symptoms were omitted due to being poor predictors of remission (Sakurai et al., 2013); (3) psychomotor activity was also omitted as it does not appear to be a consistent symptom of MDD (Hieronymus et al., 2019); (4) a question about self-blaming emotions was added; and (5) assessment intervals were changed from biweekly to weekly, as 1-week intervals are more sensitive to change (Fok and Henry, 2015). Similar to the PHQ-9, each item is rated from 0, *not at all*, to 3, *nearly every day*.

*The Generalized Anxiety Disorder Assessment* (GAD-7) (Spitzer et al., 2006) is a brief 7-item self-administered patient questionnaire that is employed to screen for and assess the severity of generalized anxiety disorder. Items ask individuals to estimate the severity of their symptoms over the past 2 weeks on a 4-point Likert Scale ranging from 0, *not at all*, to 3, *nearly every day*. Scores of 10 and 15 represent the cut-off points for moderate and severe anxiety, respectively. The GAD-7 has been reported to have strong criterion validity for identifying potential cases of GAD (Spitzer et al., 2006) and higher scores on the scale have been found to correlate with more functional impairment (Ruiz et al., 2011).

*The Positive and Negative Affect Schedule* (PANAS) (Watson et al., 1988) is a self-report questionnaire, which consists of two 10-item scales that assess positive and negative affect. Items are rated on a 5-point scale, ranging from 1, *not at all*, to 5, *very much*.

The PANAS has been validated in several languages in both western and non-western countries (Gaudreau et al., 2006; Pandey and Srivastava, 2008). It has also shown great reliability and validity in the general population, as well as in clinical samples (Watson et al., 1988; Díaz-García et al., 2020). Notably, the scale has been demonstrated to be sensitive to changes in affectivity, thus can be employed to estimate the change resulting from an intervention (Díaz-García et al., 2020).

*The Rosenberg Self-esteem Scale* (Rosenberg, 1965) is a widely used 10-item self-rated instrument that estimates global self-worth by assessing both negative and positive feelings of self-worth and self-acceptance. Items include statements such as “*I feel that I have a number of good qualities*” rated on a 4-point Likert scale ranging from strongly agree to strongly disagree. Total scores below 15 indicate low self-esteem. The validity and reliability of the scale has been tested in numerous settings and in various populations (Park and Park, 2019; Lee, 2009).

*The Standardized Assessment of Personality—Abbreviated Scale* (SAPAS) (Moran et al., 2003) is a short and simple 8-item questionnaire intended to screen for personality disorders. Each item corresponds to a yes/no question such as “*Do you normally lose your temper easily?*.” Scores of 3 or more on this screener correctly identified the presence of DSM-IV personality disorder in 90% participants. This questionnaire was included to explore whether some participants might be suffering from an undiagnosed personality disorder and to determine the most common personality profiles in the sample.

*The Mood Disorder Questionnaire* (MDQ) (Hirschfeld et al., 2000) is a brief screening instrument for bipolar disorder employed in primary care settings. It consists of 13 questions plus additional items assessing functional impairment and simultaneity of symptoms. Scores over 7 with at least moderate impairment may indicate an underlying bipolar disorder. The scale has been reported to possess good specificity and sensitivity: it can correctly identify 7 out of 10 patients suffering from a bipolar disorder and correctly screen 9 out of 10 patients without the disorder (Hirschfeld et al., 2000).

*The Patient Health Questionnaire 9* (PHQ-9) (Kroenke et al., 1999) is a biweekly 9-item self-administered instrument that intends to assess the severity of depressive symptoms. Each item assesses one of the DSM-IV criteria for MDD and is scored from 0, *not at all*, to 3, *nearly every day*. Scores of 10 or above require further assessments as they indicate the presence of moderate to severe symptoms. The PHQ-9 is a valid and reliable measure of depression’s severity; scores ≥10 had an 88% sensitivity and specificity for MDD (Kroenke et al., 2001).

*The Life Events Questionnaire* (Brugha et al., 1985) was administered at the end of the intervention to enquire about the occurrence of major adverse life events taking place during subjects’ participation in the study. The questionnaire comprised 12 yes/no questions, such as “*you had a major financial crisis*” and one open-ended questions inviting participants to write down any serious event occurring during the last week.

#### 2.2.2 Intervention materials

Participants were directed on how to create a personally relevant film-clip during the first meeting. This clip was created on a video editing software available on their chosen device; often “Video Editor” was employed for Windows devices and “iMovie” for Mac devices. The film-clip consisted of materials collected by the participants in advance of the online session. Materials mostly included pictures, songs, videos, voice recordings and quotes. Subjects were told to choose media materials, which personally evoked self-blaming-, sadness- and longing-related feelings. To illustrate, these could include a song that they often associated with the loss of a loved one or an image of a certain event for which occurrence they blamed themselves.

Media materials collected by subjects were organized according to the following structure: each film-clip began with 3 min of self-blaming related materials, followed by 3 min of sadness-related materials and ended with 4 min of longing-related materials. It is important to note that participants were verbally instructed on how to create the video during the first online meeting and were never asked to share their screen or any of the materials with the investigator. Indeed, the study did not involve any analyses or storage of the created film-clip or any of the associated materials. Nevertheless, participants were given the option of sharing their screen with the investigator, in case they required further help or were encountering an issue that could not be solved without looking at their screen. Once the film-clip had been created, participants were asked to watch it daily for a week.

### 2.3 Procedure

Subjects who met the inclusion criteria were contacted via email to provide them with the study’s information sheet and schedule two online meetings on either Zoom or Microsoft Teams.

**Figure 1:**
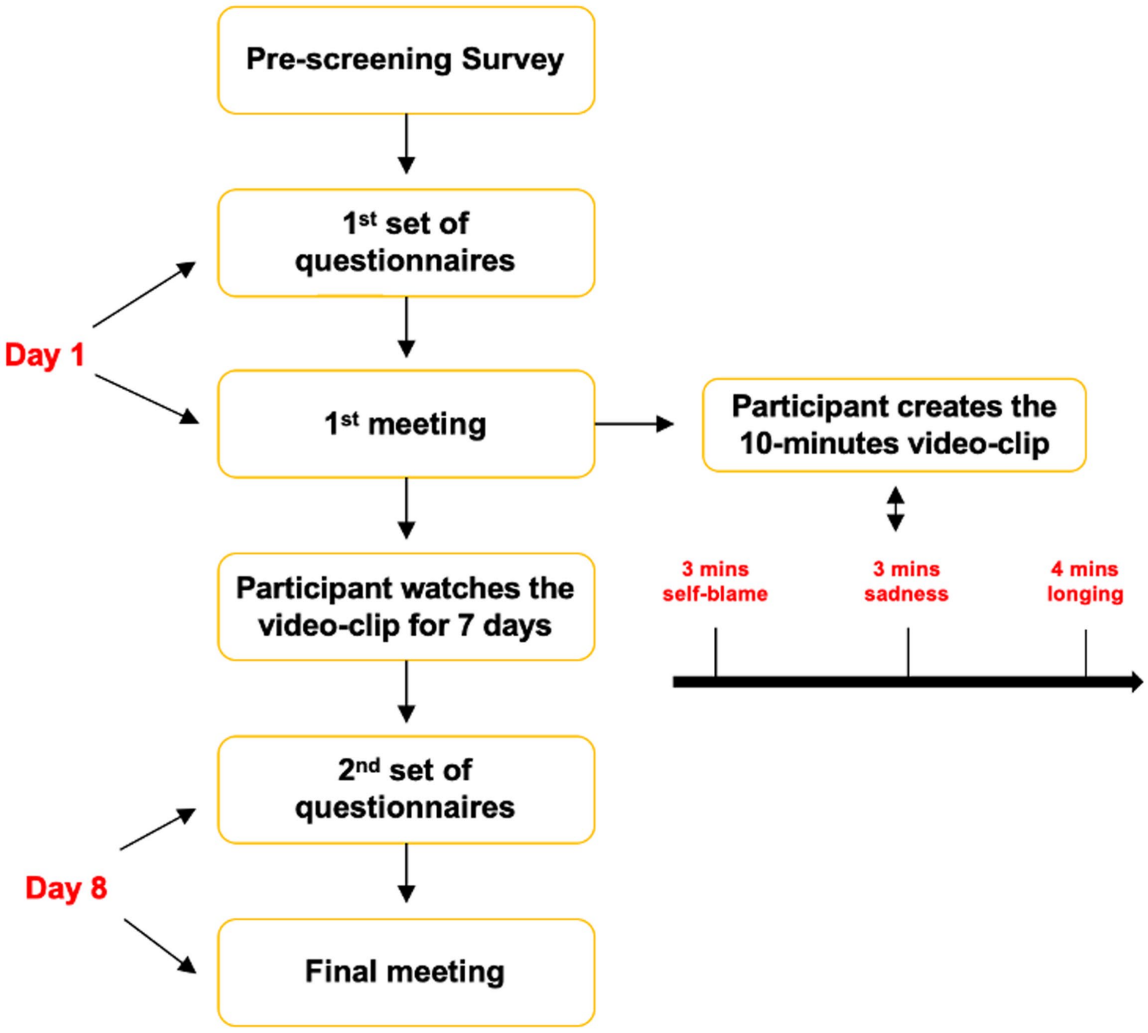
Flowchart of procedure.

As illustrated in Figure 1, in advance of the first meeting, participants were asked to fill in a set of questionnaires. Subsequently, they joined the first virtual meeting, which lasted approximately 1 h, and were invited to create the film-clip with the support of the investigator. Once the video-clip was exported, they were asked to watch it daily for a week.

After 7 days from the 1st meeting, participants were sent a different set of questionnaires. This differed from the previous one in that it included the life events questionnaire and did not comprise the SAPAS. Participants were also asked to report how many times they had watched the video during the previous 7 days. The 2nd meeting lasted 10 min and gave subjects the opportunity to share their thoughts and feedback about the intervention. At the end of the 2nd meeting, participants were emailed a £10 Amazon voucher to compensate them for their time.

### 2.4 Data analysis

All the analyses were carried out using the statistical software package SSPS for Mac (version 24), with *p* = 0.05, two-sided used as a threshold for statistical significance on our outcome measures. As our main analysis focused on pre-defined primary clinical (BDI-II) and primary non-clinical measures (Interpersonal Guilt Questionnaire), we did not correct for multiple comparisons. Significant findings on secondary measures need to be therefore considered as purely exploratory. Future adequately powered studies are therefore needed to make formal inferences using multiple comparison correction on secondary outcome measures.

According to Teare et al. (2014), with sample sizes equal or larger than 35 participants per group, accurate estimates of the standard deviation for effect sizes can be obtained. Moreover, because of the central limit theorem, with these sample sizes parametric statistics can be employed irrespective of the distribution of data. Notably, adding further participants results in less than a 10% gain in precision, which is why a sample size of 35 subjects in each group is often used for pilot and proof-of-concept studies (Frazier et al., 2016). Given that the present study employs a within-subjects design, in which data from the same participants is obtained at two distinct time points, only one group of 35 individuals was required. This design was chosen rather than a parallel group RCT to provide proof-of-concept data in an adequate sample whilst ensuring feasibility.

Scores on the questionnaires administered were compared pre- and post-intervention by running two-tailed paired-sample t-tests. For each comparison, two analyses were conducted: an intention-to-treat (ITT) analysis comprising all participants and a per-protocol analysis, which excluded participants who did not comply with the protocol, along with outliers. Outliers were defined as scores that were 2 standard deviations away from the mean.

Following these comparison analyses, further exploratory analyses were carried out on the primary outcome, to explore potential factors associated with changes in depressive scores.

## 3 Results

Most participants optimally adhered to the intervention: 35 out of 36 subjects completed both questionnaires according to the timeline, that is responding to the second one exactly 7 days after filling in the first set of questionnaires. The remaining participant filled in the post-intervention questionnaire 23 days following the initial meeting, which could lead to an inaccurate estimate of the intervention’s effect.

Fifty-six percent of participants perfectly adhered to the protocol and watched the self-created video-clip daily for 7 days. Seventeen percent watched the video on 6 days, 22% watched it on 5 days and 5% watched it only on 3 days.

With regards to the media materials employed, 44% included music in their video-clip. The number of images and videos used ranged from 1 to 30 per each video-section (e.g., self-blame related part), with an average of 8.18 ± 7.45.

Participants differed in the frequency of their self-blaming emotions at baseline: most participants reported experiencing self-blaming feelings *several days a week, for at least an hour* (47%), 8 suffered from self-blaming emotions *more than half the days, for at least an hour* (22%), 3 experienced them *every day, for an hour, but not most of the day* (8%), 4 reported self-blame only *occasionally* (11%) and the remaining 4 for *several days, but only for less than an 1 h* (11%).

Participants scores on the SAPAS ranged from 0 to 8, with an average of 2.6 ± 1.7. Fifty-three percent of them had scores of 3 or above, suggesting the possible presence of a DSM-IV Personality Disorder. According to the SAPAS, the most frequent personality profiles were “*worrier*” and “*perfectionist*.” Indeed, 69% of participants answered positively to “*are you normally a worrier?*” and 58% responded ‘yes’ to “*in general, are you a perfectionist?”*

### 3.1 Primary non-clinical outcome

**Figure 2:**
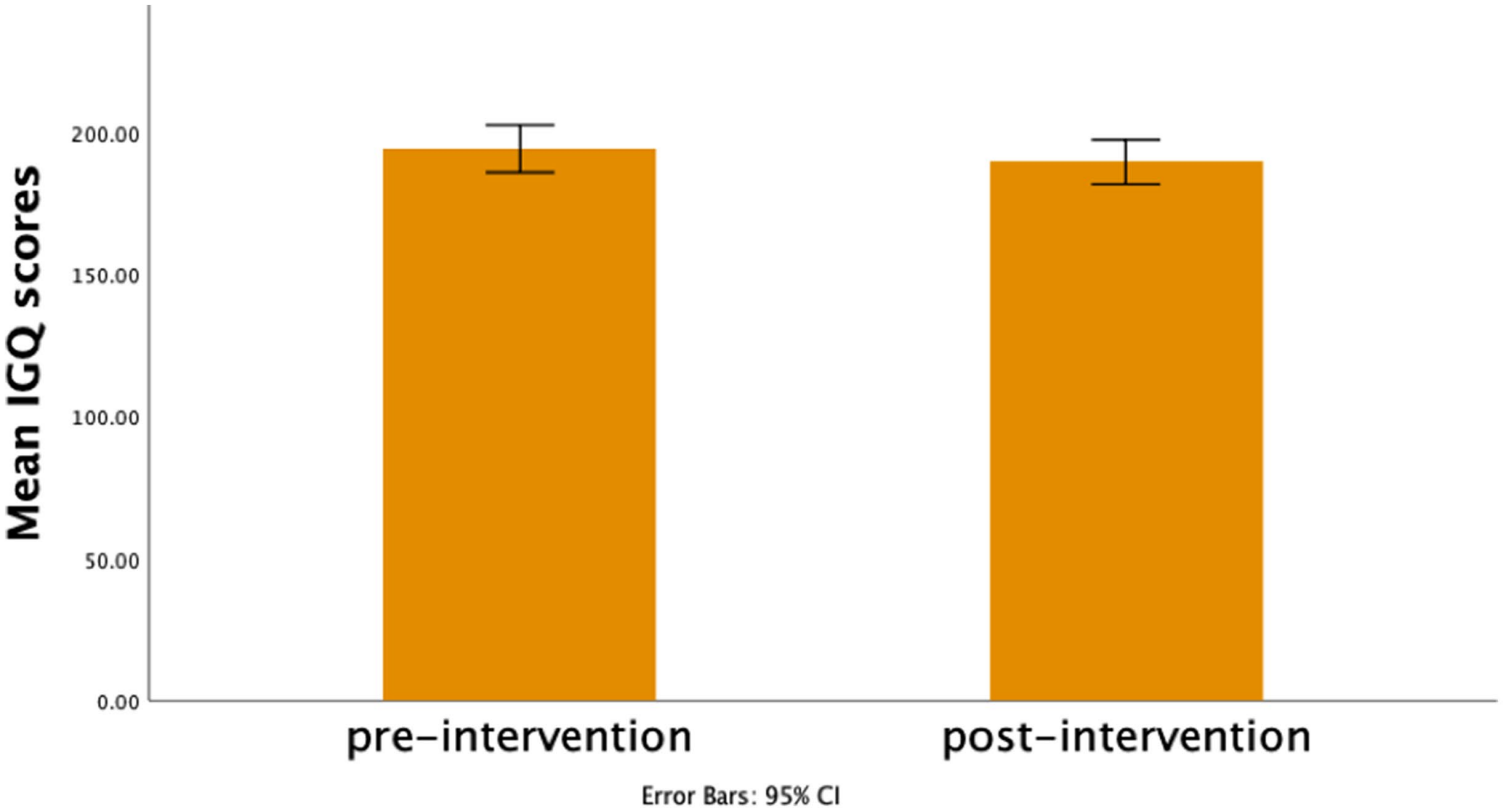
Bar Graph represents mean scores on the Interpersonal guilt questionnaire pre- and post-intervention.

Pre-intervention total scores on the IGQ-67 ranged from 151 to 244, whereas post-intervention scores’ range was 145–237. There was a slight decrease in average total scores on the IGQ-67 post intervention (*M* = 189.5 ± 23.23 vs. = 194.03 ± 24.32). Nevertheless, this reduction was not statistically significant, t = 4.53, df = 35, *p* = 0.119, CI [−1.22, 10.27], Cohen’s d = 0.27 (see Figure 2).

The analysis excluding the participant who did not adhere to the protocol, along with one outlier, led to similar results, *t* = 3.53, df = 33, *p* = 0.185, CI [−1.77, 8.83], Cohen’s d = 0.23.

To investigate whether the intervention led to changes in a particular type of guilt, a two-way repeated measures ANOVA was conducted. Scores on each subscale were averaged, as the number of items within each subscale was not equal. As confirmed by the previous paired samples t-test, there was no significant effect of time, *F*(1, 35) = 2.28, *p* = 0.140, ηp^2^ = 0.061. There was a significant main effect of subscale, *F*(3, 33) = 30.40, *p* < 0.001, ηp^2^ = 0.47. Indeed, on average, participants had higher scores on the omnipotence responsibility guilt subscale, followed by the survival guilt subscale, the separation guilt scale, and finally by the self-hate subscale. There was, however, no significant interaction between time and subscale, suggesting that the intervention did not result in a meaningful change in any type of guilt, F(3, 33) = 0.07, *p* = 0.974, ηp^2^ = 0.002.

### 3.2 Primary clinical outcome

BDI-II pre-intervention scores ranged from 0 to 32, whereas post-intervention scores ranged from 0 to 30. According to the BDI-II, scores of 30 or above are indicative of severe depressive symptoms, thus, some participants might have been suffering from an undiagnosed major depressive episode. Nevertheless, their data was included for analysis, as they had passed the inclusion criteria during the pre-screening assessment. Additionally, having data with a larger range of scores can inform on the benefit of the intervention at different levels of depression’s severity.

**Figure 3:**
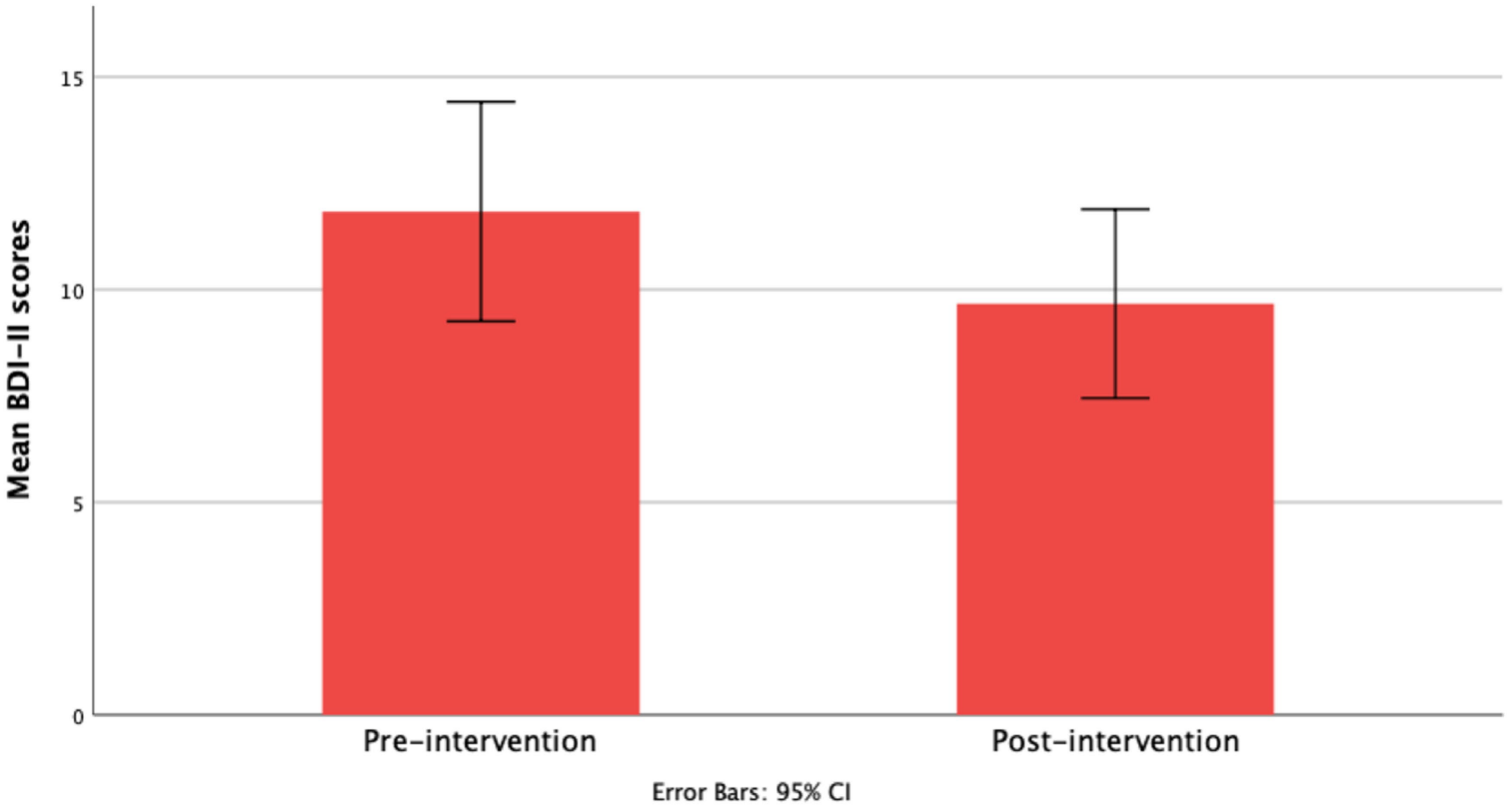
Bar graph represents mean scores on the BDI-II pre- and post-intervention.

Average BDI-II scores were lower post-intervention (*M* = 9.67 ± 6.56) compared to pre-intervention scores (*M* = 11.83 ± 7.63). This reduction was statistically significant with a small-to-medium effect size, *t* = 2.17, df = 35, *p* = 0.022, CI [0.33, 4.01], Cohen’s *d* = 0.40 (see Figure 3).

In the per protocol analysis, data from 3 participants were excluded: two were outliers and one did not adequately adhere to the intervention. Paired-sample t-test analysis suggest a significant post-intervention reduction in BDI-II scores with a moderate effect size, *t* = 2.43, df = 32, *p* = 0.002, CI [1.0, 3.85], *d* = 0.60. Thus, the significant reduction in depressive symptoms according to the BDI-II was not driven by the outliers.

### 3.3 Secondary clinical outcomes

#### 3.3.1 Anxiety symptoms

GAD-7 scores pre-intervention ranged from 0 to 16, whereas the post-intervention range was 0–13. After the intervention, average scores on the GAD-7 were lower compared to baseline (*M* = 4.50 ± 3.02 vs. 5.58 ± 4.10). This reduction in anxiety’s symptoms reached statistical significance, *t* = 1.08, df = 35, *p* = 0.023, CI [0.16, 2.01], Cohen’s *d* = 0.40.

Per-protocol analysis excluded data from 4 participants, 3 of which were outliers. Results from the paired-sample t-test analysis suggest that the reduction in anxiety’s symptoms was no longer significant, *t* = 0.719, df = 31, *p* = 0.073, CI [−0.07, 1.51], Cohen’s *d* = 0.32.

#### 3.3.2 Depressive symptoms measured by MM-PHQ-9

With regards to the MM-PHQ-9, pre-intervention scores ranged from 0 to 22, whilst post-intervention scores ranged from 0 to 18. Average scores pre-intervention were 7.17 ± 4.99, whereas post-intervention average scores were equal to 6.19 ± 4.52, suggesting a 0.98 reduction. This was, however, not significant, *t* = 0.972, df = 35, *p* = 0.054, CI [−0.02, 1.96], Cohen’s *d* = 0.33.

The per-protocol analysis, with the exclusion of 2 outliers’ data, presents different findings. Following the intervention, scores on the MM-PHQ-9 decreased from 6.45 ± 4.3 to 5.18 ± 3.11. This reduction was statistically significant with a moderate effect size, *t* = 1.27, df = 32, *p* = 0.006, CI [0.39, 2.16], Cohen’s *d* = 0.51.

### 3.4 Secondary non-clinical outcomes

#### 3.4.1 Self-esteem

Pre-intervention scores on the Rosenberg’s self-esteem scale ranged from 11 to 27, whilst post-intervention they ranged from 9 to 30. Average scores on the questionnaire pre-intervention (*M* = 19.69 ± 4.47) and post-intervention (*M* = 19.97 ± 5.07) did not appear to be different. Indeed, the paired-sample *t*-test analysis was not significant, *t* = −0.278, df = 35, *p* = 0.528, CI [−1.16, 0.607], Cohen’s *d* = 0.11.

Per-protocol analysis led to similar conclusions; pre- and post-intervention scores on the Rosenberg’s self-esteem questionnaire were not statistically significant, *t* = −0.371, df = 34, *p* = 0.401, CI [−1.26, 0.518], Cohen’s *d* = 0.14.

#### 3.4.2 Positive and negative affect

Scores on the PANAS Positive Affect scale ranged from 14 to 47 pre-intervention and from 10 to 48 post-intervention. Pre-intervention average scores on the scale were lower compared to the post-intervention ones (*M* = 26.53 ± 8.54 vs. 28.19 ± 9.83). This increase in positive affect was, nevertheless, not significant, *t* = −1.67, df = 35, *p* = 0.171, CI [−4.09, 0.75], Cohen’s *d* = 0.23.

Per-protocol analysis with the exclusion of 3 outliers’ data did not result in different findings. Indeed, the increase in positive affect remained non-significant, *t* = −2.19, df = 31, *p* = 0.066, CI [−4.53, 0.15], Cohen’s *d* = 0.34.

Finally, PANAS Negative Affect scores both pre- and post-intervention ranged from 11 to 37. Average scores were slightly lower post-intervention (*M* = 18.31 ± 6.33 vs. 19.78 ± 7.06). This reduction in negative affect did not, however, reach statistical significance, *t* = 1.47, df = 35, *p* = 0.252, CI [−1.1, 4.04], Cohen’s *d* = 0.19.

Per-protocol analysis with the additional exclusion of data from 3 outliers yielded similar findings, *t* = 2.09., df = 31, *p* = 0.099, CI [−0.42, 4.60], Cohen’s *d* = 0.30.

### 3.5 Exploratory analyses

Further exploratory analyses were only conducted for the study’s primary clinical outcome, given that an effect on the primary non-clinical outcome was not found.

To explore potential factors associated with the intervention’s effects on depressive symptoms, a new variable for the BDI-II percentage change was computed. Spearman rho correlations were then conducted to explore whether BDI-II percentage change was linked to the use of music, the amount of times the video was watched, participants’ pre-screening score on the PHQ-9 and the occurrence of significant life events. As illustrated in Table 2, none of these variables were associated with BDI-II percentage change scores to a significant degree.

**Table 2:** Potential predictors of depressive symptom changes.

Variables	1	2	3	4	5
1. BDI-II % change	–				
2. Music (yes/no)	−0.016	–			
3. Number of days watched	0.014	0.036	–		
4. Pre-screening PHQ-9	−0.232	0.082	0.043	–	
5. Life events after baseline	0.158	0.092	0.065	0.179	-

Spearman rho correlation coefficients were computed for baseline variables as potential predictors of change in depressive symptoms over the 1 week study period on the BDI-II = Beck Depression Inventory-II. PHQ-9 = Patient Health Questionnaire-9. This was an exploratory analysis and so no multiple comparison correction was carried out.

## 4 Discussion

This proof-of-concept study aimed to assess the benefits of a novel psychological intervention in reducing self-blaming feelings and depressive symptoms. With the use of emotion induction methods, as well as repetition and active thinking about self-blame-related memories, this intervention was expected to transform dysfunctional and over-generalized blame into more adaptive emotions, such as sadness and longing, which, in turn, would result in decreased depressive symptoms, as well as an overall increase in positive outcomes, such as self-esteem and positive affect.

Consistent with the study’s hypothesis, 1 week of the intervention was associated with a significant reduction in depressive symptoms, as evidenced by a decrease in scores on the BDI-II post-intervention. This remained true even after controlling for outliers and participants’ adherence. This is in line with the finding that interventions targeting self-blame can have a positive effect on general depressive symptoms (Toussaint et al., 2017). As previously discussed, this intervention prompted participant to think and face stimuli that purposefully elicited self-blaming emotions instead of avoiding or withdrawing from these memories and situations. The fact that this resulted in decreased depressive scores supports the finding that avoidance action tendencies have a detrimental impact on individuals’ mental health (Haskell et al., 2020). In addition, linking their self-blaming emotions to specific images, memories and situations, might have aided in making this feeling more context-dependent, thus, tackling their appraisal biases (Moors and Fischer, 2019; Montalti and Mirabella, 2023).

Further exploratory analyses to investigate which factors could be associated with changes in depressive symptoms revealed that the intervention’s effect was not linked to the employment of music or how many times during the week participants had watched the video, neither to negative life events nor participants’ pre-screening depressive scores. This might suggest that the benefits of the intervention are specific to its underlying mechanisms, such as prompting participants to actively think about uncomfortable memories and feelings (e.g., self-blame), along with emotion induction processes. Given that this is a self-guided intervention, more flexibility and creativity when creating the video clip would make such an intervention more accessible and more entertaining for patients. In particular, considering the simplicity of creating such film-clips, patients might be more willing to take part in the intervention.

Whereas the ITT analysis did not reveal a meaningful reduction in depressive scores as measured by the MM-PHQ-9, the per-protocol analysis revealed a moderate effect of the intervention. This is in line with the improvements in MDD symptoms observed on the BDI-II. It is, however, important to take into account the ITT analysis and discuss potential factors that might have led to the difference in findings between the BDI-II and MM-PHQ-9. ITT analyses allow investigators to draw unbiased conclusions regarding the effectiveness of an intervention (McCoy, 2017). In a clinical setting, it is likely that some patients will not optimally adhere to a protocol, thus, it is important to determine the effectiveness of treatments independently from individuals’ characteristics and adherence.

Divergence in findings might be due to the difference in sensitivity to change between the two scales. Although the MM-PHQ-9 would be expected to be more sensitive to change considering its weekly interval, the BDI-II captures a wider range of depressive symptomatology, thus, identifying improvements on certain aspects that might have not been assessed by the former instrument. Considering that this was a proof-of-concept analysis on participants with low depressive symptoms, a reduction in MM-PHQ-9 scores, although non-significant according to the ITT analysis, is promising.

The intervention did not, however, lead to meaningful changes in self-blame, as assessed by the IGQ-67. This could be due to the nature of the instrument: the IGQ-67 could be defined as a static or dispositional assessment, and, as such, may not be suitable for capturing weekly changes in blame. In fact, beliefs such as “*I do not deserve other people’s respect or admiration*” suggest that such feelings are deeply rooted in how a person perceives themselves and the surrounding world. Although these can change with time, a one-week intervention might not be sufficient to provoke meaningful alterations to individuals’ beliefs. Questionnaires that assess weekly changes in self-blame by, perhaps, asking people about the thoughts and feelings they have experienced in the previous few days (e.g., *I felt guilty for disagreeing with a loved one this week*), may be more adequate. Unfortunately, to date, assessments of self-blame are scarce and their sensitivity to change has not been evaluated.

With regards to the secondary outcomes, a reduction of anxious feelings was observed post-intervention in the ITT, but not in the per-protocol analysis. Although scores on the other assessments did not meaningfully change, comparison of means pre-and post-intervention suggest that they moved in the expected direction. As an example, on average, positive affect scores were higher post-intervention, whereas negative affect scores were lower.

Considering that this is a proof-of-concept study, the focus should be on the feasibility of the intervention. Notably, this novel psychological intervention can easily be carried out and accessed by a greater number of patients. Indeed, it does not require the involvement of a professional, it is delivered online, the costs are minimal and can be fully self-guided.

Importantly, adherence rates were excellent; except for one participant, everyone adhered to the 8 days schedule, although a couple of subjects watched the self-created video clip less than 5 times per week. Additionally, drop-out rates were relatively low as well: out of 39 participants recruited, only 7%, that is 3 people, dropped out following the 1st online meeting.

Crucially, the safety of the intervention in this sample was confirmed by the absence of adverse events: no participants reported experiencing significant levels of distress throughout the intervention and although support was provided, it has not been requested by any subject. This could suggest that the intervention did not result in negative outcomes, however, it does not erase the possibility that some participants might not have felt comfortable reaching out for help. This was, however, unlikely, as no increase was observed on any of the negative outcomes, including anxiety, negative affect, depressive symptoms and self-blame and no decrease was found on the positive outcomes, that is on positive affect and self-esteem.

It is important to mention that milder symptoms of depression have been previously reported to be more difficult to treat (Hieronymus et al., 2019). People with non-severe depression are less likely to experience meaningful improvements relative to those suffering from severe depression; indeed, even when treated with antidepressants, a positive association between severity and efficacy is observed (Hieronymus et al., 2019). This could be accounted for by the absence of much room for improvement in patients with milder symptoms. Considering that participants in this current study displayed none to very mild depressive symptoms at baseline, as they were screened for mood disorders, observing an improvement in their depressive scores was even more challenging. To illustrate, experiencing troubles with sleep or loss of interest from time to time is normal and does not entail clinical impairment, thus, these experiences are not necessarily indicative of depressive symptoms and, as such, cannot be tackled by treatments for MDD. Additionally, the recruitment of participants who experienced self-blaming emotions but did not meet the clinical threshold for depression might have resulted in a sample of patients with anxiety, which was not screened for. Nevertheless, the benefits observed following 1 week of intervention in this sample are encouraging and, hence, provide a good basis to testing this novel psychological intervention in a clinical sample.

Notably, although participants were screened for depressive symptoms before recruitment, some of them exhibited high scores on the BDI-II and MM-PHQ-9 at the beginning of the intervention, which is usually indicative of severe depression. Nevertheless, given that they had met the inclusion criteria, their data was included and further provided us with a rationale for implementing this intervention in a sample of MDD patients. In fact, given that no adverse outcomes were observed, such findings suggest that this intervention appears to be safe at different levels of depression symptoms’ severity. However, further studies are warranted to corroborate the benefits of this intervention in a clinical sample.

As with any other treatment, this intervention might not be suitable for everyone. Given its underlying mechanisms, particularly the focus on engaging participants in active thinking about uncomfortable and possibly negative memories, this intervention might be more beneficial for patients who tend to avoid or suppress negative thoughts and emotions. Indeed, it has been pointed out that thought suppression contributes to the maintenance of intrusive cognitions, such as those observed in MDD (Wegner et al., 1987; Davies and Clark, 1998). Whereas, talking or writing about unwanted thoughts, thus facing one’s intrusive cognitions, resulted in a sense of relief and reduced the frequency of these thoughts (Pennebaker, 1997; Pennebaker et al., 1990). Depending on the severity of an individuals’ tendency to suppress negative thoughts, actively facing one’s negative memories can be more or less beneficial.

Other factors that can influence the benefits of this novel psychological intervention are a person’s ability to voluntarily experience mental imagery, that is aphantasia (Zeman et al., 2015), and to feel emotions conveyed by music (Taruffi et al., 2017). Indeed, considering that this intervention relies on mood-induction processes, it is likely to benefit people who are sensitive to emotions portrayed in images or communicated through music.

### 4.1 Limitations

It is worth acknowledging that the current study presented some important limitations apart from not being randomized against a control intervention. First, all instruments administered relied on participants’ self-report. Specifically, there was no objective method in place to determine how many times during the week participants had watched the film clip. Thus, it cannot be concluded with certainty that they had adhered to the intervention, as some might have over-reported the days on which they watched the video. Moreover, as investigators did not have access to the media materials employed by participants, they could not determine whether the images and songs used were adequate and fulfilled the criteria. Nevertheless, given the observed benefits following 1 week of intervention, it is likely that some flexibility with regards to the media items included in the film clip can be granted, as what appears to be crucial is the meaning of these materials.

As previously briefly mentioned, this study was limited by the questionnaires administered. Instruments such as the IGQ-67 and the Rosenberg self-esteem questionnaire may, indeed, not be adequate for the assessment of weekly changes following an intervention. However, measures such as the BDI-II and the MM-PHQ-9, which are sensitive to temporal changes, are also limited in their ability to capture the effect of this weekly intervention. Indeed, the BDI-II aims to assess bi-weekly changes in depressive symptoms, whereas the MM-PHQ-9 is a weekly assessment. Considering that this psychological intervention is one weeklong, retrospective assessment with such intervals capture the emotions and symptoms experienced throughout the whole period of the intervention and do not provide a clear picture of a person’s change in mood following their participation in the study. This is well illustrated by participants’ feedback during the final virtual meeting: indeed, almost everyone reported experiencing more intense feelings of self-blame and sadness on the 1st few days of the intervention, however, these feelings slowly attenuated towards the end of the week and, in many cases, resulted in more positive and inspiring thoughts and emotions. Instruments with shorter time intervals would, therefore, be more suitable.

Alternatively, the intervention could be delivered for longer periods, such as two or 3 weeks. Questionnaires could, therefore, be administered pre-intervention, halfway through the intervention and a few days following the end of the intervention. Thus, providing a better overview of the alterations in individuals’ feelings, perceptions, mood and clinical symptoms brought forward by their participation in this study.

Unfortunately, the study’s sample was predominantly female, therefore, limiting the generalizability of these findings to a male population. The benefits of the intervention might indeed be specific to certain individual characteristics of our sample and may not be observed in a different population.

Additionally, the study’s findings might have been subject to repetition bias, considering that the same instruments have been administered at two different time points. However, this is unlikely, given that changes in questionnaires’ scores did not follow the same trend: on certain measures scores increased post-intervention, while on others they decreased.

Notwithstanding these limitations, results from the present study are encouraging and provide a good rationale for assessing this intervention in a clinical sample. Importantly, the simple, straightforward, and economic nature of the intervention would make it accessible to a larger number of patients. It could, therefore, represent a novel alternative to current self-guided interventions, such as computerized CBT. Its strengths also rely on its use of implicit learning strategies, which are less demanding relative to CBT’s mechanisms, and, thus, would perhaps lead to higher acceptance rates among MDD patients.

This intervention could also be provided to individuals who are on the waiting list for therapy, or as a 1st step of treatment to encourage patients to actively engage with psychological therapy. Indeed, as reported by several participants, towards the end of the intervention, they felt more pro-active and motivated, as well as more likely to develop a new and more accepting perspective on their life and on what had been previously negatively affecting them.

Future studies are, nevertheless, warranted to assess whether similar benefits can be achieved in a clinical sample and to determine whether such benefits are maintained once the intervention is over. Additionally, a control group could also be introduced to ensure that the observed benefits are specific to the intervention, and not due to non-specific factors, such as talking to the investigator. A control group could, as an example, be asked to watch an unrelated video, such as a 10-min clip from a movie or could include media materials that are not specifically relevant to the participant.

## 5 Conclusion

The detrimental impact of depression on patients’ quality of life stresses the importance of developing novel psychological interventions, which can be easily accessed by those in need. The present study provides a promising alternative to current interventions that could contribute to the reduction of depressive symptoms by tackling self-blaming emotions. Through emotion-induction methods, as well as active thinking about negative experiences, this intervention resulted in decreased depression and anxiety-related scores in just 1 week.

Further studies are necessary to determine whether this intervention can be beneficial in clinical samples.

## Data Availability

The raw data supporting the conclusions of this article will be made available by the authors, without undue reservation.
